# Data report on career adaptability, personal value, and motivational interview among at-risk Chinese college students

**DOI:** 10.1016/j.dib.2023.108982

**Published:** 2023-02-14

**Authors:** Tingyu Gu, Xiaosong Gai, Yuan Wang, Fanli Jia

**Affiliations:** a School of Psychology, Northeast Normal University, No. 5268, Renmin Street, Changchun, Jilin Province, China; b Department of Psychology, Seton Hall University, 400 S Orange Ave, South Orange, NJ, USA

**Keywords:** Career construction, Peer coaching, Intervention, Counseling

## Abstract

The dataset provided here was partially associated with a published article on career adaptability [1]. The data set included 343 college freshmen who had difficulties in career decision-making. A self-report questionnaire on career adaptability (concern, control, curiosity, confidence), personal values (materialistic values, self-transcendence values, self-enhancement values), and demographic information was administered to all participants. In addition, a pre-selection of low career adaptability was performed. These participants scored below the 27th percentile in career adaptability. The career adaptability was administrated again two months later. We divided the data into two groups (intervention and control) and two time points (pre-test and post-test). Researchers can use the data to explore the relationship among career adaptability, personal values, and demographic information, as well as to compare interventions on career adaptability.

Specifications table

**Table:** 

Subject	Psychology

Specific subject area	Applied Psychology – Career Intervention
Type of data	Quantitative data
How data were acquired	Questionnaire survey (see supplementary material)
Data format	Raw, Analyzed, Filtered (descriptive and inferential statistics)
Description of data collection	Data was collected from students majoring in foreign languages at six universities in Jilin Province. Three hundred and ninety-one students were invited through email by local collaborators from these universities. In total, 343 first-year college students completed the Career Adapt-Abilities Scale-China form [2], the Personal Values Scale [3], and demographic data online. A response rate of 87.72% was obtained from the invitation. The motivational interviews were conducted with the at-risk students who scored low on career adaptability. The Career Adapt-Abilities Scale was administered two months after the motivational interview as a post-test.
Data source location	Northeast Normal University, Jilin Province, China.
Data accessibility	Repository name: Mendeley Data Data identification number: DOI: 10.17632/xhp7fdpmmm.2 Direct URL to data: https://data.mendeley.com/datasets/xhp7fdpmmm/2
Related research article	X. Gai, T. Gu, Y. Wang, F. Jia, 2020. Improving career adaptability through motivational interview among peers: An intervention of at-risk Chinese college students majoring in foreign language, J. Vocat. Behav. 138, 103762. https://doi.org/10.1016/j.jvb.2022.103762.

## Value of the data

• The raw data of self-reported career adaptability, personal values, and demographic information across six Chinese universities.
• The data set includes pre-test and post-test scores of career adaptability including career control, concern, curiosity, and confidence.
• Analysis of variance, multiple regression, latent profile model, and structural equation modeling can be used to analyze the data.
• By coding demographic information, it may be possible to compare the training effects of motivational interviewing with similar studies in parallel samples of different genders, ages, and socioeconomic status (occupations of parents and educational degrees).

## Objective

In this data report, additional values are added to the published article in the following ways: 1. The data set here provides additional demographic attributes including universities, gender, and SES which are not fully explored in the published article. 2. Parental information, including educational backgrounds and occupations of the mothers and fathers, is included in the data set. There is a potential for researchers to examine how parental education and occupation affect the careers of their children. 3. The data set here contains personal values (materialistic values, self-transcendence values, self-enhancement values), which are not included in the published article. Researchers can obtain information about how college students’ different values system relates to their career adaptability and their demographic characteristics.

## 1 Data description

The data provided here was partially used in a published article on career adaptability [1].

**Fig. 1:**
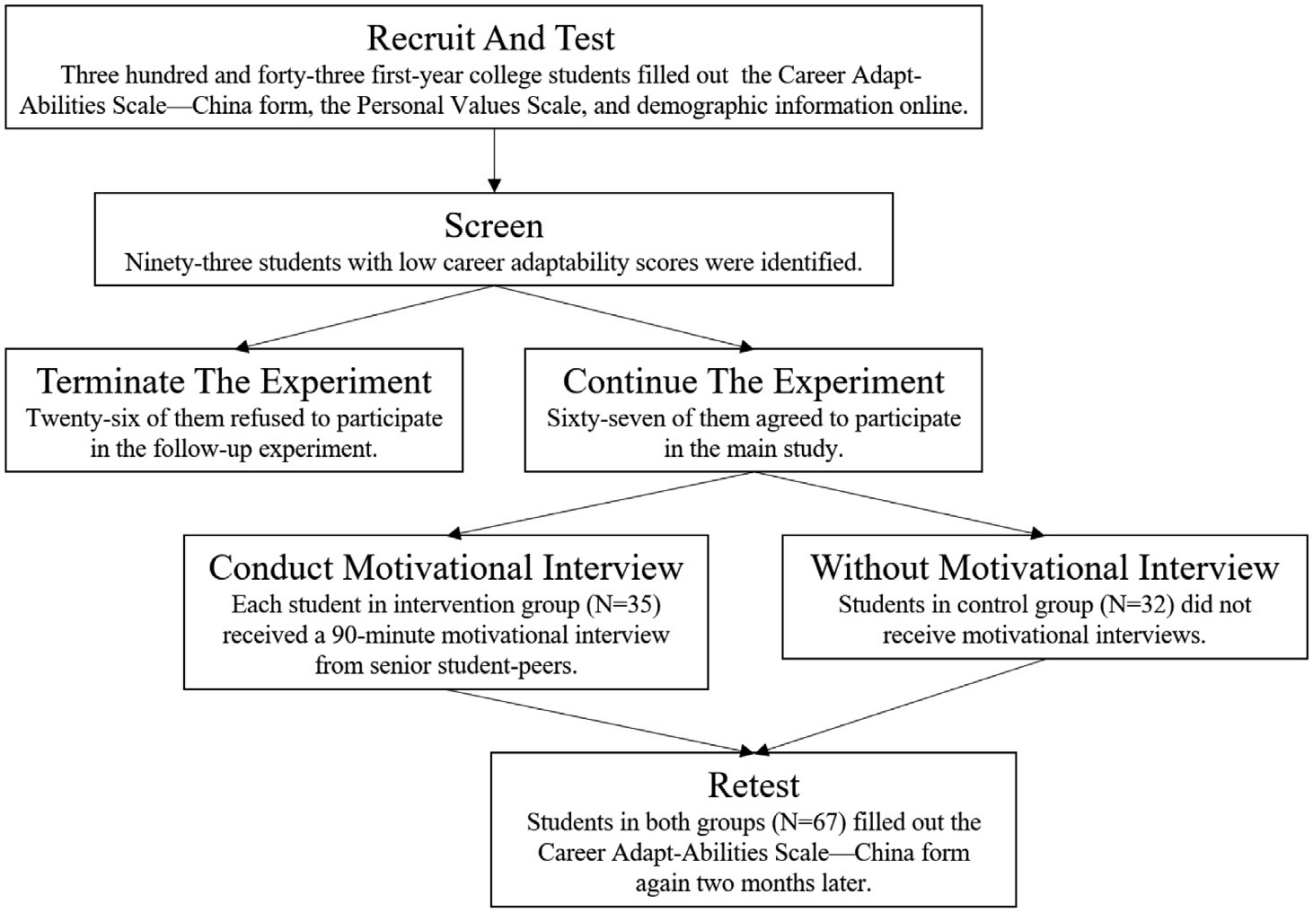
Recruitment procedure

Names of the six universities were unidentified using random codes in order to maintain confidentiality. The data contains self-reported responses on career adaptability (concern, control, curiosity, confidence), personal values (materialistic values, self-transcendence values, self-enhancement values), and demographic information (gender, and age, father's current occupation, mother's current occupation, father's highest level of education, and mother's highest level of education) of 343 college freshmen majoring in foreign languages across six universities in Jilin Province, China. Participants who scored below the 27th percentile on career adaptability were asked if they would be interested in participating in the follow-up study. A total of sixty-seven participants were present. A random assignment was performed to place the selected participants in an intervention group (N=35) or in a control group (N=32). The participants in the intervention group received motivational interviews within one week of being assigned to the group. A detailed description of motivational interviewing is provided in the published article associated with the data [1]. Participants in the control group did not receive anything during this week. The post-test was conducted two months after the experimental manipulation. The variables included in the data file are summarized in Table 1. Fig. 1 summarizes the recruitment procedure and experimental design.

**Table 1:** Data file.

Variable	Variable description	Variable type	Variable labels
Identify	Whether participated in the follow-up study	Nominal	0 = was not selected; 1 = was selected but not participated in the follow-up experiment; 2 = was selected and participated in intervention group; 3 = was selected and participated in control group
School	School	Nominal	Universities were unidentified using random codes: 1 = CUT; 2 = JNU; 3 = JISU; 4 = CHSC; 5 = NENU; 6 = TNU
Gender	Gender	Nominal	1 = Male; 2 = Female
Age	Age (YY, e.g., 18)	Numeric	17 = 17 years … 23 = 23 years
FatherOcc	Father's current occupation	Numeric	1 = Temporary or unemployed workers; 2 = Self-employed workers; 3 = Operators; 4 = agriculture, forestry, animal husbandry, fishery and water conservancy workers; 5 = Commercial or service workers; 6 = Clerks; 7 = professional and technical personnels; 8 = Persons in charge of state organs, armed forces, companies or institutions
MotherOcc	Mother's current occupation	Numeric	1 = Temporary or unemployed workers; 2 = Self-employed workers; 3 = Operators; 4 = agriculture, forestry, animal husbandry, fishery and water conservancy workers; 5 = Commercial or service workers; 6 = Clerks; 7 = professional and technical personnels; 8 = Persons in charge of state organs, armed forces, companies or institutions
FatherEdu	Father's highest level of education	Numeric	1 = Some primary school studies, or completed primary school or junior high school; 2 = Completed high school or technical secondary school; 3 = At least completed college diploma
MotherEdu	Mother's highest level of education	Numeric	1 = Some primary school studies, or completed primary school or junior high school; 2 = Completed high school or technical secondary school; 3 = At least completed college diploma
SES	Total score of parental occupations and educational degrees	Numeric	As calculated
Concern1 … Concern6 a	Career concern of total sample	Scale	As reported
Control1 … Control6 b	Career control of total sample	Scale	As reported
Curiosity1 … Curiosity6 c	Career curiosity of total sample	Scale	As reported
Confidence1 … Confidence6 d	Career confidence of total sample	Scale	As reported
Concern	Average score of career concern of total sample	Numeric	As calculated
Control	Average score of career control of total sample	Numeric	As calculated
Curiosity	Average score of career curiosity of total sample	Numeric	As calculated
Confidence	Average score of career confidence of total sample	Numeric	As calculated
Adaptability	Average score of four subscales of the Career Adapt-Abilities Scale—China form	Numeric	As calculated
Materialistic1 … Materialistic7 e	Materialistic values of total sample	Scale	As reported
SelfTranscendence1 … SelfTranscendence5 f	Self-transcendence values of total sample	Scale	As reported
SelfEnhancement1 … SelfEnhancement5 g	Self-enhancement values of total sample	Scale	As reported
Materialistic	Average score of materialistic values of total sample	Numeric	As calculated
SelfTranscendence	Average score of self-transcendence values of total sample	Numeric	As calculated
SelfEnhancement	Average score of self-enhancement values of total sample	Numeric	As calculated
PostConcern1 … PostConcern6 a	Post-test of career concern of the at-risk subsample	Scale	As reported
PostControl1 … PostControl6 b	Post-test of career control of the at-risk subsample	Scale	As reported
PostCuriosity1 … PostCuriosity6 c	Post-test of career curiosity of the at-risk subsample	Scale	As reported
PostConfidence1 … PostConfidence6 d	Post-test of career confidence of the at-risk subsample	Scale	As reported
PostConcern	Average post-test score of career concern of the at-risk subsample	Numeric	As calculated
PostControl	Average post-test score of career control of the at-risk subsample	Numeric	As calculated
PostCuriosity	Average post-test score of career curiosity of the at-risk subsample	Numeric	As calculated
PostConfidence	Average post-test score of career confidence of the at-risk subsample	Numeric	As calculated
PostAdaptability	Average post-test score of four subscales of the Career Adapt-Abilities Scale—China form	Numeric	As calculated

abcd Subscales of the Career Adapt-Abilities Scale—China form, 6 items for each subscale, refer to supplementary material.

efg Subscales of the values scale, 7 items for materialistic values subscale, 5 items for self-transcendence values subscale, 5 items for self-enhancement values subscale, refer to supplementary material.

## 2 Experimental design, materials and methods

The study was approved by the Ethics Review Committee from the institution of the first author. Researchers have obtained permission to use the following measures with citations.

For the selection and testing of participants as well as for the effectiveness of motivational interviewing, a Career Adaptability Scale-China form [2] was used. A previous study confirms the structure of the Chinese version [4]. The scale consists of six items related to career concerns (e.g., “Thinking about what my future will be like”), six items about career control (e.g., “Making decisions by myself”), six items about career curiosity (e.g., “Looking for opportunities to grow as a person”), and six items about career confidence (e.g., “Performing tasks efficiently”). A five-point Likert format (1 = “not strong” to 5 = “very strong”) was used. Cronbach's α coefficients can be calculated for the subscales and the combined scale. The Cronbach's α coefficients for all items and for each subscale were greater than 0.80, indicating a high level of reliability.

The Personal Values Scale [3] was used to examine personal values of total sample. This scale contains seven items about materialistic values (e.g., “Getting money, status and other material wealth is an important thing in life”), five items about self-transcendence values (e.g., “The meaning of my life is to contribute to the happiness of others”), five items about self-enhancement values (e.g., “I strive to master more knowledge and skills”). A five-point Likert format (1 = “completely disagree” to 5 = “completely agree”) was used. For the whole sample (N = 343), Cronbach's α coefficients for the whole scale and each subscale exceeded 0.70, indicating reasonable reliability.

Demographic information included gender, age, and SES (parental occupation and educational degree). Parental occupations and educations were classified into various levels (see Table 1). SES can be calculated based on both factors (e.g., father's/mother's current occupations, and father's/mother's highest education level.)

## Ethics statements

The study was carried out in accordance with the Declaration of Helsinki and was approved by the Ethics Review and Research Committee at the School of Psychology from Northeast Normal University – Protocol # 201707. All participants provided their informed consents before conducting the study. For minor-aged participants (under 18 years old), informed consents from their parents were also obtained before data collection.

## CRediT authorship contribution statement

**Tingyu Gu:** Conceptualization, Methodology, Project administration, Formal analysis, Investigation, Writing – original draft, Writing – review & editing. **Xiaosong Gai:** Conceptualization, Methodology, Validation, Investigation, Resources, Writing – original draft, Writing – review & editing, Supervision, Funding acquisition. **Yuan Wang:** Formal analysis, Investigation. **Fanli Jia:** Conceptualization, Methodology, Writing – original draft, Writing – review & editing, Supervision.

## Declaration of Competing Interest

The authors declare that they have no known competing financial interests or personal relationships that could have appeared to influence the work reported in this paper.

## Data Availability

Raw Data: Career Adaptability, Personal Value, and Motivational Interview Among At-risk Chinese College Students (Original data) (Mendeley Data)

## Appendix Supplementary materials

Image, application 1
