# Supplementary material for: Data report on career adaptability, personal value, and motivational interview among at-risk Chinese college students
Source: Data Brief. 2023 Feb 14;47:108982. doi: 10.1016/j.dib.2023.108982 (PMC9989514; doi:10.1016/j.dib.2023.108982)
Supplement: Supplementary file 1 [file mmc1.doc]

Descriptive statistics on demographic variables can be obtained from functions in Excel file.

<https://data.mendeley.com/datasets/xhp7fdpmmm/2>

In addition, researchers can transform the format of the data file to Statistical Package for the Social Sciences (SPSS), a statistical software that is often used in Social Science research. Syntax of descriptive analysis for demographic variables is provided:

*Mean and Standard Deviation:*

DESCRIPTIVES VARIABLES=Gender Age FatherOcc MotherOcc FatherEdu MotherEdu SES

/STATISTICS=MEAN STDDEV.

*Frequency:*

FREQUENCIES VARIABLES= Gender Age FatherOcc MotherOcc FatherEdu MotherEdu SES

/ORDER=ANALYSIS.

Correlation analysis among career adaptability, personal values, and demographic variables can also be conducted. Syntax of correlation analysis is provided:

*CORRELATIONS*

/VARIABLES=Concern Control Curiosity Confidence Adaptability Materialistic SelfTranscendence SelfEnhancement Gender Age FatherOcc MotherOcc FatherEdu MotherEdu SES

/PRINT=TWOTAIL NOSIG

/MISSING=PAIRWISE.

Repeated measure ANOVAs can be conducted to examine the influence of the motivational interviewing intervention on the career adaptability and its subcomponents (career concern, career control, career curiosity, career confidence). Syntax of 2 × 2 repeated measure ANOVA on the career adaptability is provided as an example:

GLM Adaptability PostAdaptability BY Identify

/WSFACTOR=time 2 Polynomial

/METHOD=SSTYPE(3)

/POSTHOC=Identify(LSD)

/PLOT=PROFILE(time*Identify)

/EMMEANS=TABLES(Identify) COMPARE ADJ(BONFERRONI)

/EMMEANS=TABLES(time) COMPARE ADJ(BONFERRONI)

/EMMEANS=TABLES(time*Identify) COMPARE(time) ADJ(LSD)

/EMMEANS=TABLES(time*Identify) COMPARE(Identify) ADJ(LSD)

/PRINT=DESCRIPTIVE ETASQ OPOWER HOMOGENEITY

/CRITERIA=ALPHA(.05)

/WSDESIGN=time

/DESIGN=Identify.
